# Giant Ependymoma Removal via a Skipped Myelotomy Technique

**DOI:** 10.7759/cureus.44511

**Published:** 2023-09-01

**Authors:** Haydar Gok, Suat Erol Celik, Kivanc Yangi, Salih B Kartal, Arzu Dobral

**Affiliations:** 1 Neurological Surgery, Prof. Dr. Cemil Tascioglu City Hospital, Istanbul, TUR; 2 Neurological Surgery, Tokat Public Hospital, Tokat, TUR; 3 Pathology, Prof. Dr. Cemil Tascioglu City Hospital, Istanbul, TUR

**Keywords:** long segment myelotomy, myelotomy, skipped myelotomy, intramedullary tumors, spinal ependymoma

## Abstract

Intramedullary ependymomas should be treated with surgical resection. Different surgical techniques are described for these tumors, such as skipped and long-segment myelotomies. A 31-year-old male patient with a giant thoracic spinal cord ependymoma extending from the level of T5 to T10 was operated on with a skipped myelotomy technique. Although the patient had urinary incontinence and muscle weakness in both legs, the patient’s complaints were nearly completely resolved in the fourth postoperative month. Operating with the smallest possible myelotomy has given us preferable results; however, more studies are needed to hypothesize the superiority of this technique over conventional myelotomy.

## Introduction

Intramedullary spinal cord tumors (IMSCT) are rare and comprise less than 4% of central nervous system tumors and 30% of intradural spinal neoplasms. The most common tumor types are ependymomas and astrocytomas [1]. Intramedullary ependymomas are solitary tumors of the spinal cord that arise from the ependymal cells lining the central canal. They cause diffuse cord enlargement over several segments and arise most commonly in the cervical spine [2,3].

Many patients with intramedullary ependymomas present with back pain and dysesthesias without sensory loss because intramedullary ependymomas cause symmetrical expansion around the central canal, interrupting the spinothalamic tract's crossing fibers [2]. On MRI, ependymomas are generally hypointense on T1-weighted images and hyperintense on T2-weighted and fluid-attenuated inversion recovery images and enhance homogeneously with gadolinium contrast material. Approximately 65% have associated cysts, mainly when cervical regions are involved [2,4,5].

Intramedullary ependymomas are treated by surgical resection, and gross-total resection can be accomplished without significant morbidity. However, significant neurological deficits may occur in such cases [6-8]. Alternative operative techniques are needed in order to avoid these complications. This study emphasizes that skipped myelotomy may be an alternative to long-segment myelotomy. On the other hand, postoperative radiation therapy is recommended for patients with anaplastic ependymoma, regardless of whether gross total resection is achieved. Chemotherapy is not routinely used to treat ependymomas [6-8].

## Case presentation

**Figure 1:**
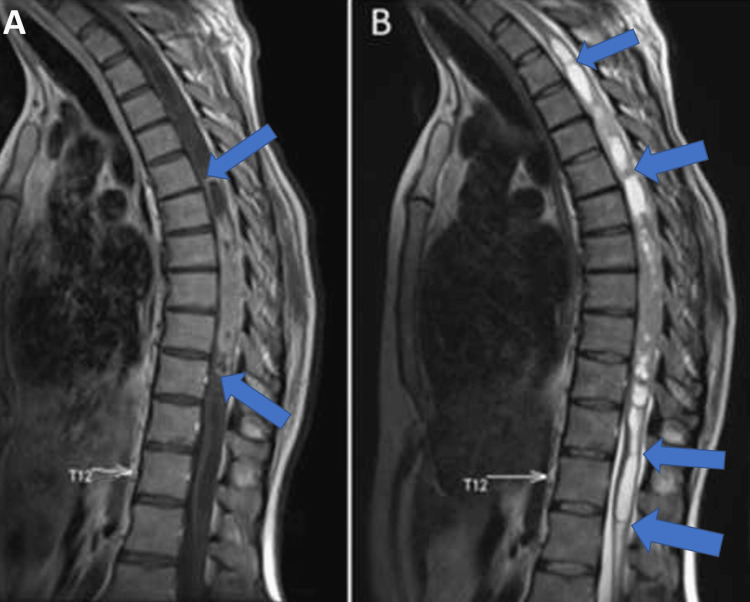
Preoperative MRI of the thoracic spine showing the lesion and the syrinx cavity (A) T1-weighted sagittal scan with gadolinium enhancement showing intramedullary homogeneously enhanced mass extending from the vertebral level of T5 to T10. Blue arrows indicate the borders of the lesion. The white arrow indicates the vertebral level of T12. (B) T2-weighted sagittal scan showing the syrinx cavity (blue arrows). The white arrow indicates the vertebral level of T12.

**Figure 2:**
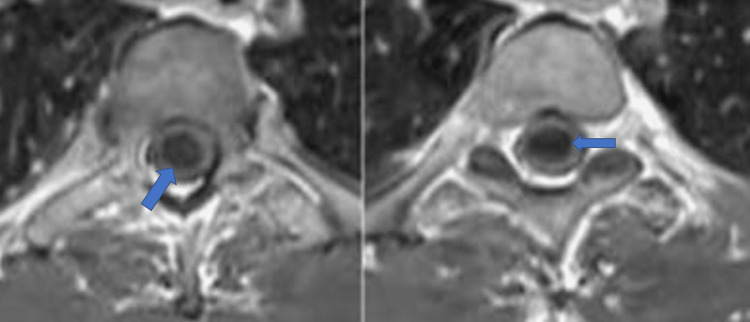
Preoperative MRI of the thoracic spine indicating the syrinx cavity Preoperative T1-weighted axial scan showing the syrinx cavity (blue arrows).

A 31-year-old male patient was admitted to our department with complaints of right leg pain, numbness in both legs, and difficulty walking. He stated that his difficulty in walking started about seven months ago, and he decided to come to the hospital when his pain became unbearable. In muscle strength testing, 4/5 muscle strength was noted in the extension and flexion of the hip and both knees. The remaining muscle strengths were found to be normal. The straight leg raise test was positive on the right side. Bilateral deep tendon reflexes were hyperactive. Babinski's sign was positive bilaterally. Contrast-enhanced MRI of the thoracic spine showed a large solid mass extending from the vertebral level of T5 to T10 and located in the intramedullary compartment (Figure 1). There was also large syringomyelia above and below the tumor, almost along the spinal canal (Figures 1-2).

**Figure 3:**
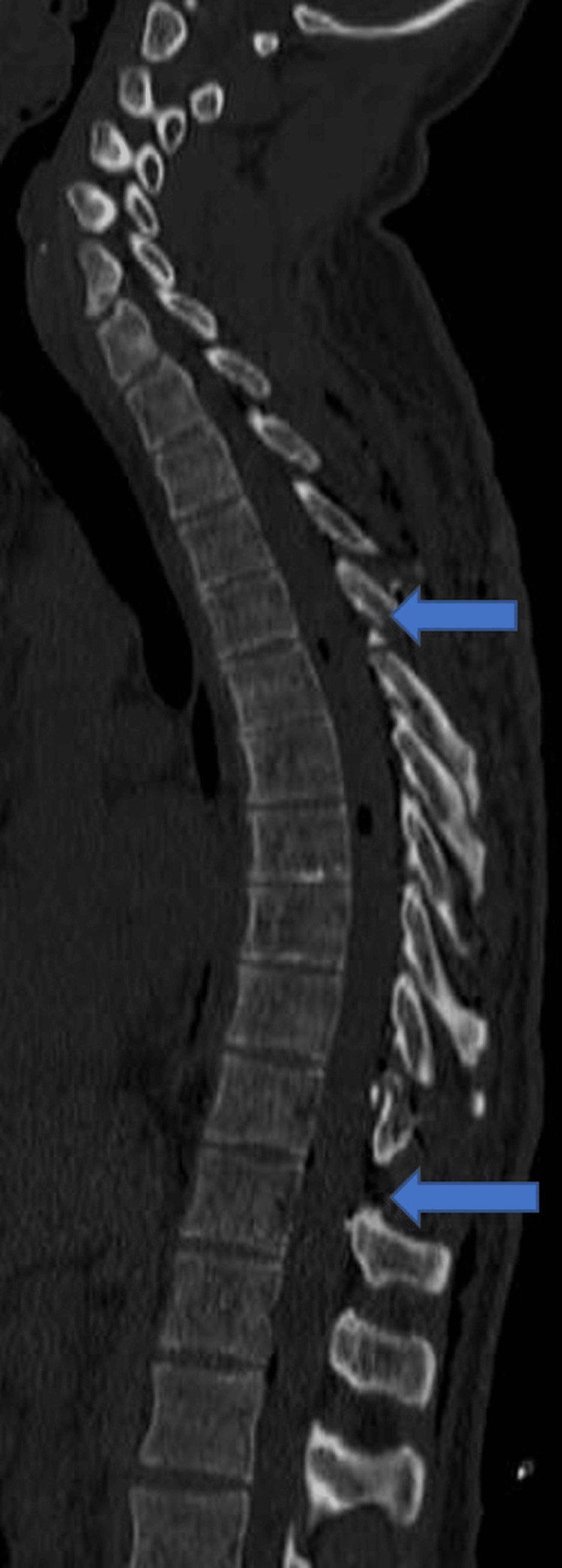
Postoperative CT scan of the thoracic spine showing the borders of laminoplasty Postoperative sagittal CT scan of the thoracic spine showing that laminoplasty was performed. The blue arrows indicate the borders of laminoplasty.

**Figure 4:**
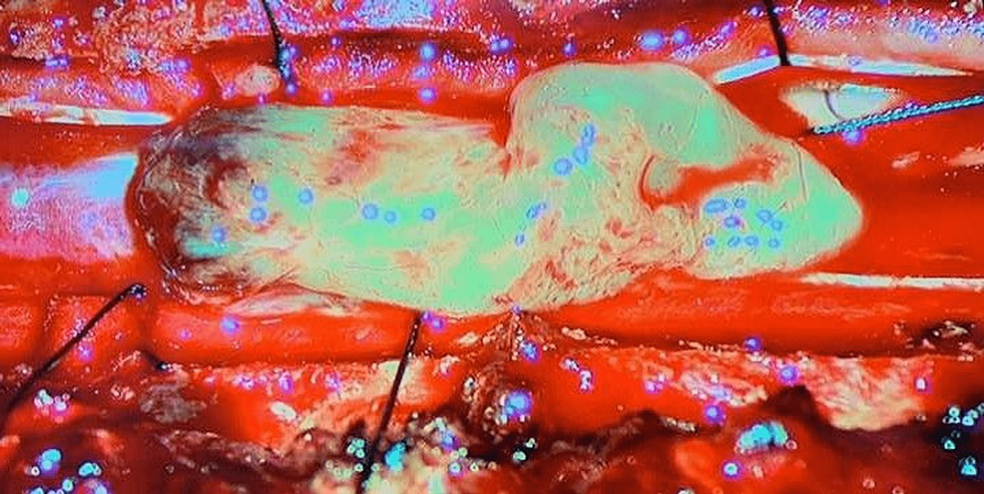
Intraoperative image of the lesion The intramedullary mass was stripped from the glial tissue with blunt dissection and gross-totally excised. It was red-purple and had a medium consistency.

**Figure 5:**
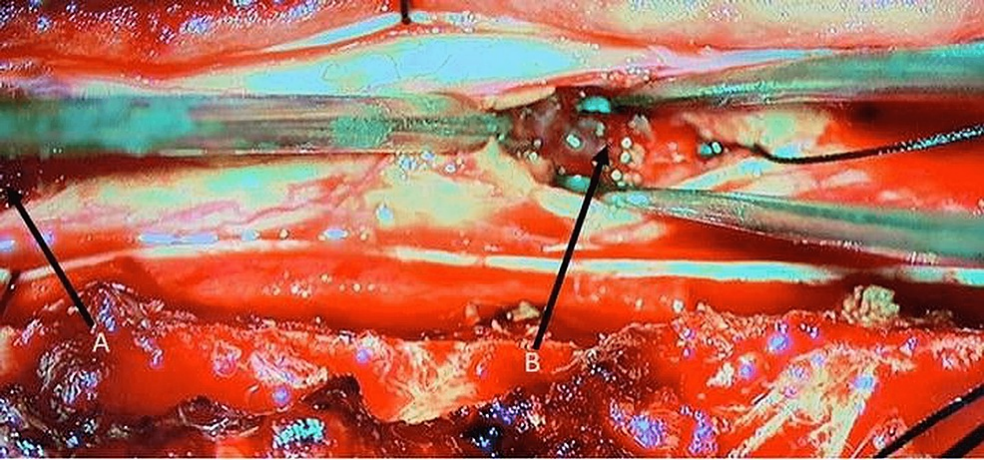
Intraoperative picture of the spinal cord showing the skipped myelotomy technique The mass was removed by the skipped myelotomy technique. The locations are indicated by the black arrows. A and B are the areas where the myelotomy was performed.

**Figure 6:**
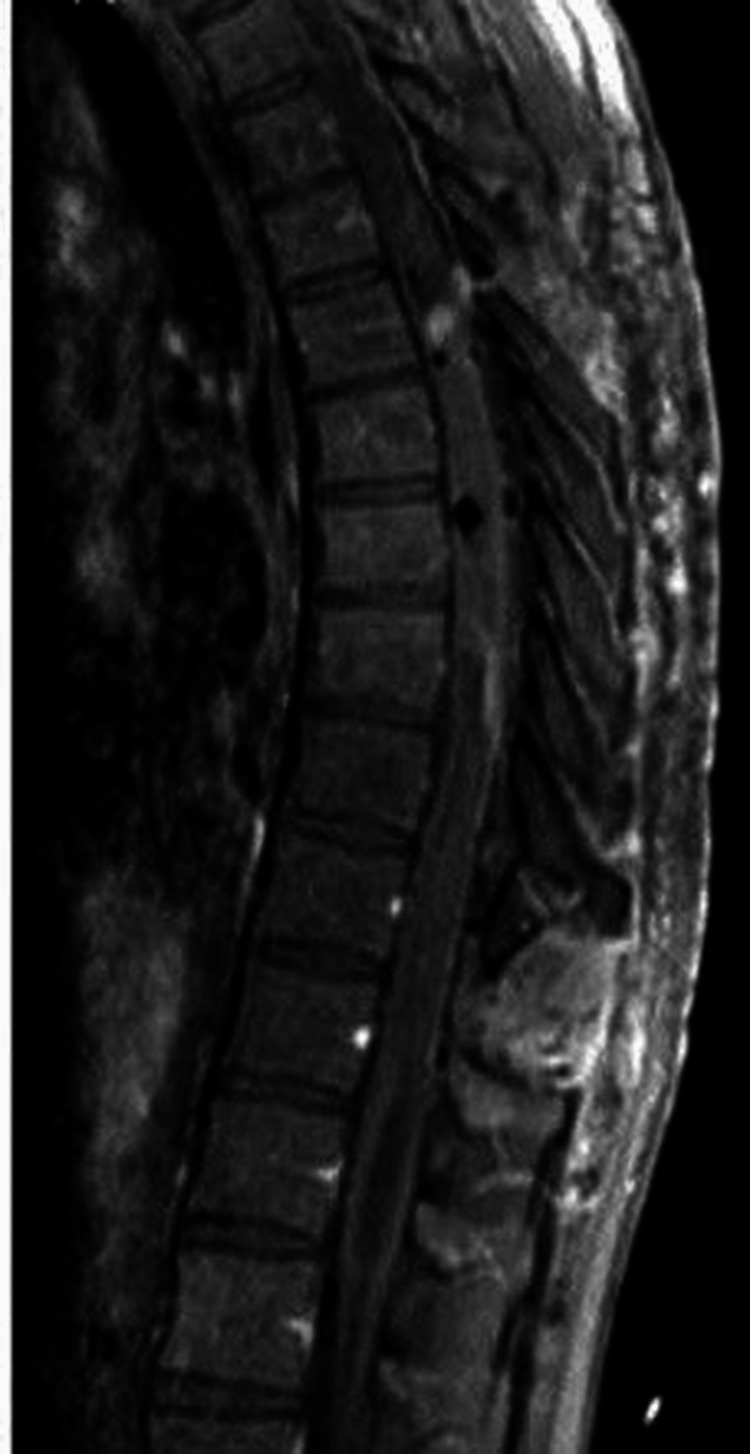
Postoperative MRI of the thoracic spine showing complete removal of the lesion Postoperative T1-weighted sagittal MRI of the thoracic spine with gadolinium enhancement showing that the lesion was gross-totally excised.

After the patient's consent, he underwent the operation under general anesthesia. A posterior midline incision and subperiosteal dissection of the muscles were performed until complete laminar exposure was achieved. The laminoplasty technique was performed under intraoperative neuromonitoring with somatosensory evoked potentials (SEPs) and motor evoked potentials (MEPs). After confirmation by fluoroscopic imaging, the laminae (from one level above to one below the level of the lesion) were cut using a bone excavator (ultrasonic surgery bone cutting kit, Dmetec, Gyeonggi, South Korea) and removed as a whole (Figure 3). The dura mater was opened longitudinally and anchored with sutures laterally. Then, the arachnoid mater was opened under the surgical microscope, and the skipped myelotomy technique was performed throughout the elongated tumor. Initially, a 3 cm midline myelotomy was performed from cranial to caudal. A 2 cm long area under the first myelotomy was preserved. A 3 cm myelotomy with 2 cm preserved areas was continued caudally. The intramedullary mass was stripped from the glial tissue with blunt dissection and gross-totally excised by three separate myelotomies. The mass was red-purple and had medium consistency (Figures 4-5). After removing the mass, amplitude reduction and latency increases were detected for the right lower extremity's MEPs records. Intravenous methylprednisolone was administered promptly after the significant reduction of amplitudes. Postoperative contrast-enhanced MRI of the thoracic spine showed that the gross-total resection was accomplished (Figure 6).

**Figure 7:**
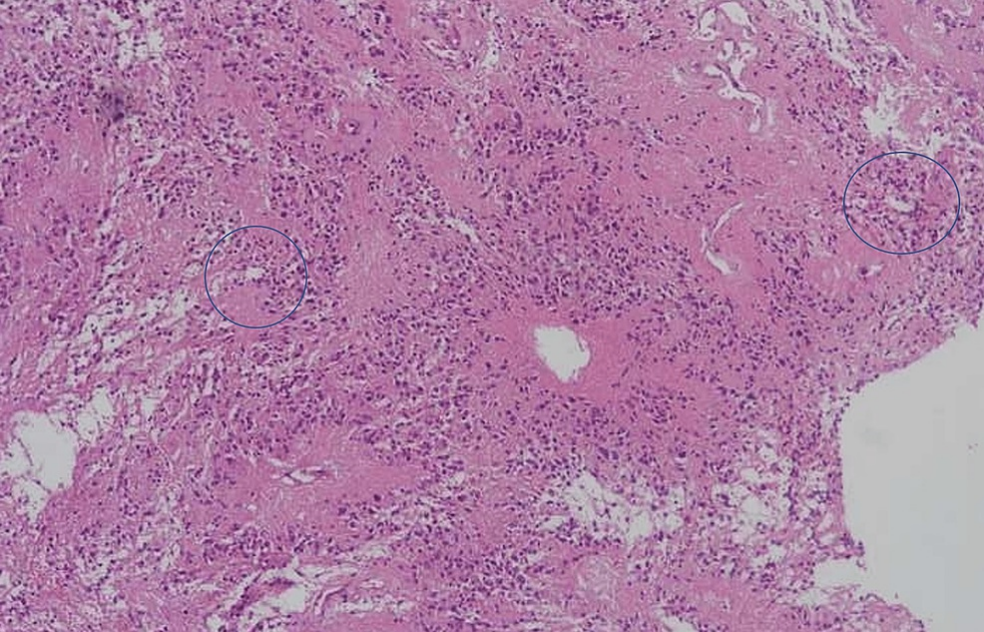
Pathologic specimen picture, H&E, x100 Ependymal rosettes composed of columnar cells arranged around fibrillary zones (blue circles).

**Figure 8:**
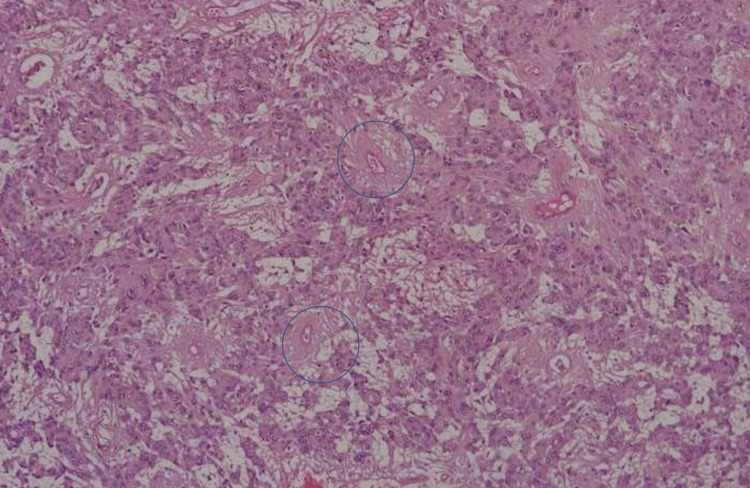
Pathologic specimen picture, H&E, x100 Ependymal pseudorosettes characterized by radially arranged tumor cells around blood vessels creating perivascular anucleate zones (blue circles).

**Figure 9:**
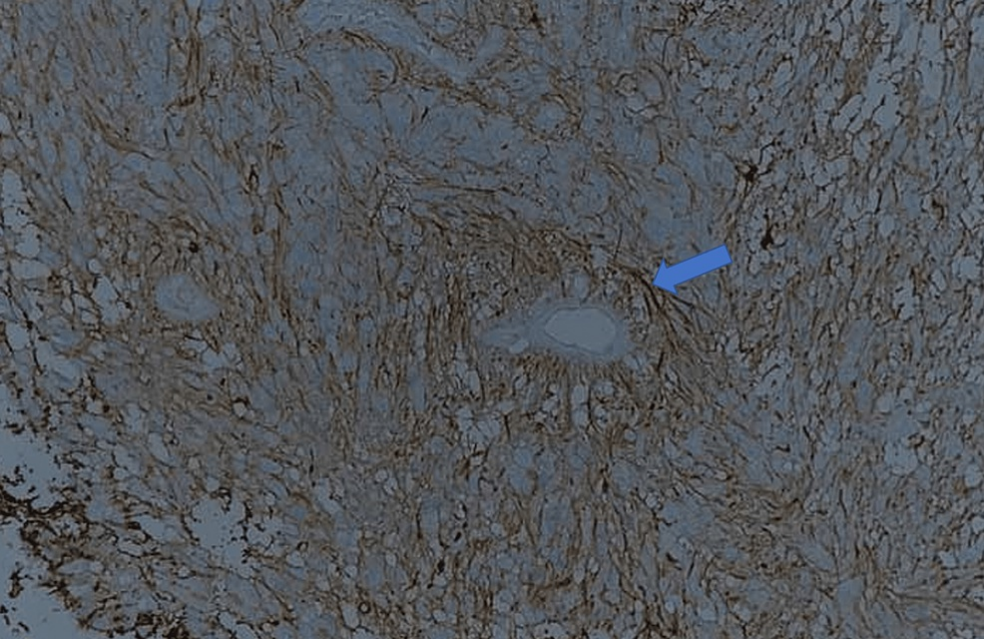
Pathologic specimen picture, GFAP, x200 Immunohistochemistry with GFAP showing the fibrillary processes in the perivascular anucleate zones (blue arrow).

**Figure 10:**
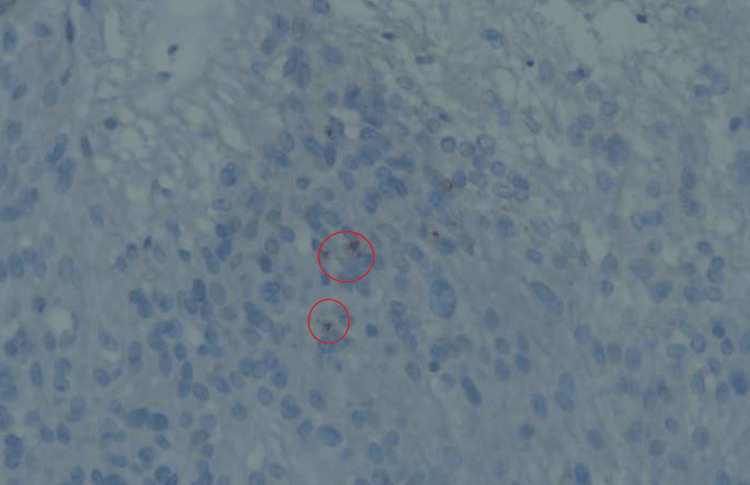
Pathologic specimen picture, EMA, x400 EMA immunostaining is seen in dot-like cytoplasmic structures (red circles).

**Figure 11:**
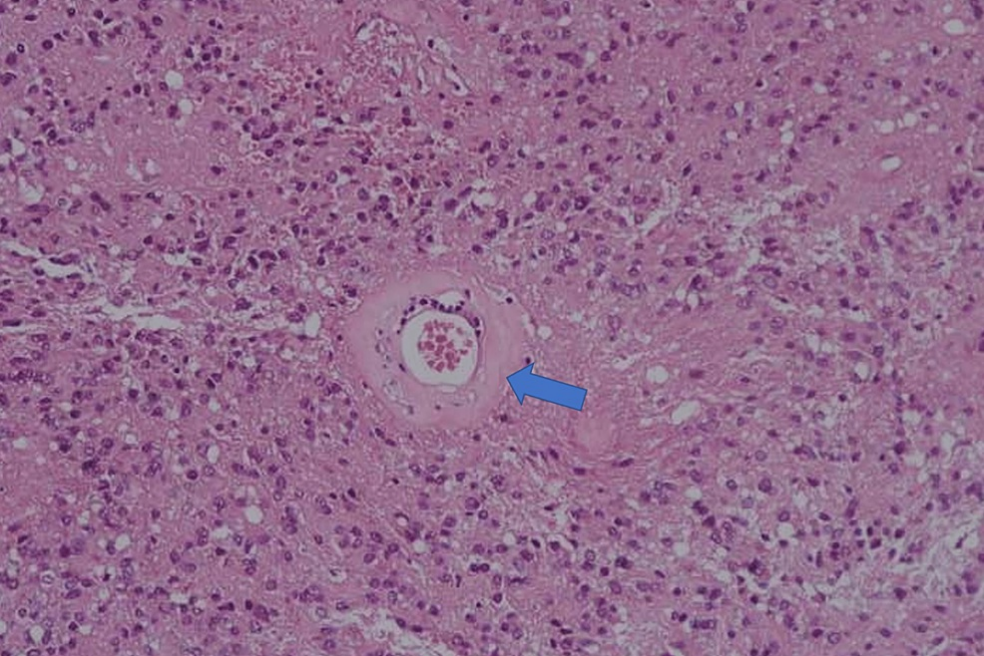
Pathologic specimen picture, H&E, x200 Prominent hyalinization of tumor vessels (blue arrow) (H&E, x200)

There was a decrease in muscle strength after the surgery (1/5 reduction for the right lower extremity and 2/5 reduction for the left lower extremity). Furthermore, the patient complained of urinary incontinence. The early rehabilitation program was started for the patient, and he underwent rehabilitation service on the eighth postoperative day. Currently, he can stand up and walk without support. The patient's urinary incontinence complaint was almost entirely resolved. Pathology reports resulted in ependymoma WHO Grade II (GFAP and S100 positive, EMA sparse dot painting, Ki-67 index <1%) (Figures 7-11).

## Discussion

Ependymomas are the most common intramedullary tumors in adults [9]. They may rarely have exophytic features, mimicking an extramedullary lesion. They are most common in the middle ages. The cervical region is the most common level of true intramedullary occurrence; however, 40% of intradural ependymomas arise from the filum terminale [10,11]. They account for 40% to 60% of IMSCT in adults and 16% to 35% in children. More than 90% of spinal cord ependymomas have a benign pathology. They are slowly growing lesions that tend to compress adjacent spinal cord parenchyma rather than infiltrate it. Although unencapsulated, these neuroglia-derived tumors are usually well-circumscribed and do not usually infiltrate adjacent spinal cord tissue, which allows for total resection in 70% to 100% of cases [4].

The most effective treatment method is surgery. Complete resection is the primary goal of surgery to reduce tumor recurrence and, whenever possible, to preserve neurological function [12]. About 50% of spinal cord ependymomas extend along three or more vertebral levels; the remaining 50% are smaller than three vertebral levels [13]. As in our case, "giant" spinal ependymomas are very rare [14]. Hyperesthesia, diminished proprioception, paresthesias, and incoordination of gait are the complications of the myelotomy procedure [15]. Such complications are more likely to occur in patients who require long-segment myelotomy, such as ours. Therefore, we tried to perform the smallest possible myelotomy. While doing this, we ensured it was large enough to work comfortably without damaging the cord.

The patient’s postoperative neurological function was worsened compared to the preoperative state. This might result from the traction of the posterior column, microtrauma resulting in transient edema, or vascular damage. Patients with spinal cord ependymomas in the thoracic spinal cord are more likely to develop neurological deficits after resection because of the limited blood supply in this region and the narrow spinal canal [16]. However, we do not think of vascular injury for this patient since neurological deficits were recovered within four months.

According to our knowledge, most patients experience the resolution of their somatosensory deficits within a few months [17-20]. Thus, after four months of physiotherapy, our patient can walk and do his daily work without support. As a result, we are sharing the information that skipped myelotomy may be an alternative surgical technique in giant IMSCT. It may help us protect the spinal cord from damage. The weakness of our study is that we have tried this method only in one case. Further studies are needed with more cases to prove this method’s efficacy and superiority over long-segment myelotomy.

## Conclusions

The skipped myelotomy technique should be considered the smallest possible myelotomy during the preoperative planning of large intramedullary tumors. In our case, the best possible outcome has been accomplished regarding neurological deficits. However, more studies with more cases are needed to hypothesize the superiority of this technique over long-segment myelotomy and other techniques.
